# Manganese-based hollow nanoplatforms for MR imaging-guided cancer therapies

**DOI:** 10.1186/s40824-022-00275-5

**Published:** 2022-07-06

**Authors:** Shuang Liang, Guangfu Liao, Wenzhen Zhu, Li Zhang

**Affiliations:** 1 Department of Radiology, Tongji Hospital, Tongji Medical College, Huazhong University of Science and Technology, Wuhan, 430030 China; 2 Engineering Research Center of Nano-Geomaterials of Ministry of Education, China University of Geosciences, Wuhan, 430074 China; 3 School of Science, Harbin Institute of Technology (Shenzhen), Shenzhen, 518055 China

## Abstract

Theranostic nanoplatforms integrating diagnostic and therapeutic functions have received considerable attention in the past decade. Among them, hollow manganese (Mn)-based nanoplatforms are superior since they combine the advantages of hollow structures and the intrinsic theranostic features of Mn^2+^. Specifically, the hollow cavity can encapsulate a variety of small-molecule drugs, such as chemotherapeutic agents, photosensitizers and photothermal agents, for chemotherapy, photodynamic therapy (PDT) and photothermal therapy (PTT), respectively. After degradation in the tumor microenvironment (TME), the released Mn^2+^ is able to act simultaneously as a magnetic resonance (MR) imaging contrast agent (CA) and as a Fenton-like agent for chemodynamic therapy (CDT). More importantly, synergistic treatment outcomes can be realized by reasonable and optimized design of the hollow nanosystems. This review summarizes various Mn-based hollow nanoplatforms, including hollow Mn_x_O_y_, hollow matrix-supported Mn_x_O_y_, hollow Mn-doped nanoparticles, hollow Mn complex-based nanoparticles, hollow Mn-cobalt (Co)-based nanoparticles, and hollow Mn-iron (Fe)-based nanoparticles, for MR imaging-guided cancer therapies. Finally, we discuss the potential obstacles and perspectives of these hollow Mn-based nanotheranostics for translational applications.

**Graphical Abstract:**

Mn-based hollow nanoplatforms such as hollow Mn_x_O_y_ nanoparticles, hollow matrix-supported Mn_x_O_y_ nanoparticles, Mn-doped hollow nanoparticles, Mn complex-based hollow nanoparticles, hollow Mn-Co-based nanoparticles and hollow Mn-Fe-based nanoparticles show great promise in cancer theranostics.
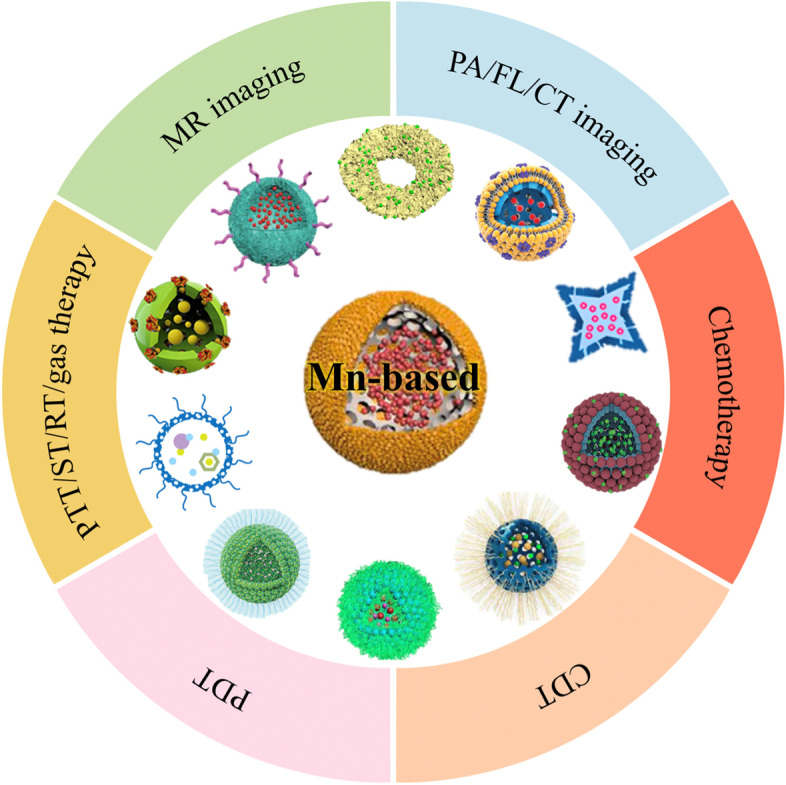

## Introduction

Cancer has always been considered one of the deadliest diseases that threatens human life, and the number of cases is increasing year by year [1–4]. Theranostic nanoplatforms that integrate diagnostic and therapeutic units have proven to be significant for cancer treatment in the past decade [5–7]. Among the imaging approaches in clinical use, magnetic resonance (MR) imaging, developed based on the nuclear magnetic resonance (NMR) principle, is more attractive due to its non-invasiveness and potent spatial resolution, especially for soft tissue detection [8–10]. With the rapid advances in nanotechnology, a variety of MR imaging contrast agents (CAs) have been utilized for improving the resolution and sensitivity during scans [11, 12]. To date, CAs have been involved in more than 40% of MR imaging examinations [13]. Generally, MR imaging CAs can be divided into two major categories based on their relaxation processes, i.e., T_1_ and T_2_ CAs. T_1_ CAs are able to shorten the longitudinal relaxation time of the surrounding water protons and increase the signal intensity of T_1_-weighted images, while T_2_ CAs shorten the transverse relaxation time of the surrounding water protons and reduce the signal intensity of T_2_-weighted images [14–17]. Paramagnetic Gd^3+^ complexes and iron oxide (IO) nanoparticles are representative commercial T_1_ and T_2_ CAs, which are beneficial to the detection of tumors [18–20].

Nevertheless, the widespread clinical application of T_2_-weighted MR imaging is hampered by confusion between the negative contrasts generated and other pathological environments, which limits diagnostic accuracy [21]. In addition, IO nanoparticles with high susceptibility distort the circumjacent magnetic field and cause blurred images [22, 23]. Taking all these factors into consideration, T_1_ CAs are more promising than T_2_ CAs for precise and high-resolution imaging [24]. However, the premature leakage of Gd^3+^ may cause systemic toxicity, and Gd^3+^ complex CAs often suffer from short blood circulation as well as nonspecific distribution [25–27]. The development of new positive CAs with all the above-mentioned issues resolved is urgently needed. Over the past 10 years, Mn-based nanomaterials such as Mn_x_O_y_ and MnS have drawn increasing interest in biomedical applications [28–32]. These nanoplatforms with passive or active targeting ability are capable of selectively accumulating at the tumor site, which leads to highly effective MR imaging after degradation in the tumor microenvironment (TME) [33, 34]. In addition, Mn is one of the necessary elements in human bodies for metabolism, and its uptake and excretion can be efficiently controlled by biological systems, resulting in low toxicity and high biosafety [35–37]. Notably, single-modal imaging with insufficient diagnostic information sometimes cannot meet the high requirements of modern medicine [38–41]. Therefore, there is increasing interest in the exploration of multi-modal imaging CAs. For instance, dual-modal T_1_- and T_2_-weighted MR imaging that integrates both positive and negative CAs merits can allow enhanced diagnosis by highlighting the anatomical details in MR images [42–44]. Moreover, the combination of MR imaging with fluorescence (FL) imaging is capable of providing complementary information, as FL imaging compensates for the inferior sensitivity of MR imaging, and in turn, MR imaging remedies the weak spatial resolution and tissue penetration of FL imaging [45–47]. Furthermore, photoacoustic (PA) imaging is a newly developed modality that incorporates the advantages of both optical and ultrasonic imaging, and much attention has been given to constructing various CAs for dual-modal MR/PA imaging [48–50].

To overcome the side effects and simultaneously improve the therapeutic outcome of conventional chemotherapy and radiotherapy (RT), various smart drug delivery systems (DDSs), especially hollow DDSs, have been explored [51–55]. The preparation strategies for high-quality hollow DDSs can be divided into two main categories: (1) sacrificial template-based methods, which exploit a variety of removable nanoparticles as hard templates (e.g., silica, polystyrene and metal-organic frameworks (MOFs)) [56–61] or soft templates (e.g., Pluronic F127/TMB and gas bubbles) [62, 63]; and (2) self-templating methods, which employ the transformation of self-generated internal solid nanoparticles to hollow structures during chemical reactions [64–68]. The former approach has been widely applied to produce various hollow nanoparticles with uniform morphology and a tuneable diameter and shell thickness, such as hollow MnO_2_ [69, 70], hollow polydopamine (PDA) [71, 72], hollow carbon [73, 74] and hollow mesoporous organosilica nanoparticles (HMON) [75, 76], whereas the relatively recently developed latter approach is considered superior owing to the simple synthetic procedures and reduced formation of chemical waste [77]. For the self-templating method, the nanoscale Kirkendall effect, galvanic replacement reaction and Ostwald ripening process are often used to prepare hollow Cu_7_S_4_ nanocrystals [78], Au−Ag@Au hollow nanostructures [79] and hollow cuprous oxide@nitrogen-doped carbon dual-shell structures [80], respectively. More importantly, DDSs with responsive diagnostic and therapeutic functions can assist in tumor detection as well as monitor drug release and treatment processes within a certain TME [60, 81]. Among the hollow TME-responsive DDSs, Mn-based nanoplatforms display tremendous promise in bioimaging, drug delivery and tumor therapy owing to their good biocompatibility, unique hollow structures and excellent physical/chemical performances [69, 82]. For instance, hollow MnO_2_ nanoparticles can rapidly respond to the TME, catalyzing intracellular hydrogen peroxide (H_2_O_2_) to produce O_2_ and concurrently depleting the overexpressed glutathione (GSH) [33, 83]. The generated O_2_ benefits additional treatment modalities, such as chemotherapy [84], RT [85], photodynamic therapy (PDT) [84], sonodynamic therapy (SDT) [86] and starvation therapy (ST) [87], while the consumption of GSH leads to redox imbalance and further improves the curative effects of reactive oxygen species (ROS)-mediated therapies [88]. Moreover, the released Mn^2+^ can serve as a good Fenton-like agent for chemodynamic therapy (CDT) and simultaneously as a CA for T_1_-weighted MR imaging [89, 90]. Furthermore, due to the degradation of hollow structures in the TME, the loaded cargoes can be released to perform diverse treatments under the guidance of MR imaging [69, 82, 91]. By reasonable and optimal design, such hollow nanosystems are also expected to realize synergistic diagnostic and therapeutic outcomes [81, 92].

**Scheme 1:**
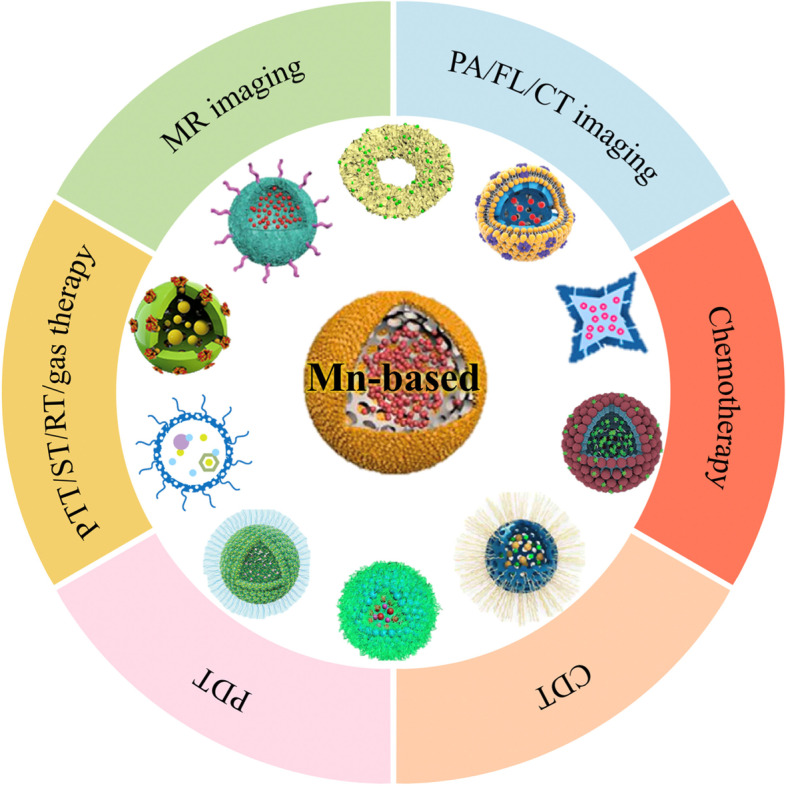
Schematic illustration of various Mn-based hollow nanoplatforms for cancer theranostics.

This review summarizes the recent progress in Mn-based hollow nanoplatforms for MR imaging-guided cancer therapies, with several sections presented according to the different nanostructures. In each chapter, the basic introduction of the corresponding hollow materials is first given, followed by a detailed summary of the applications including MR, PA, FL and computer tomography (CT) imaging as well as chemotherapy, CDT, PDT, photothermal therapy (PTT), ST, RT and gas therapy. In addition to single-magnetic-core Mn-based hollow nanoplatforms including hollow Mn_x_O_y_, hollow matrix-supported Mn_x_O_y_, Mn-doping hollow nanoparticles and Mn complex-based hollow nanoparticles, dual-magnetic-core hollow Mn-Cobalt (Co)-based nanoparticles and hollow Mn-iron (Fe)-based nanoparticles are also introduced (Scheme 1). Finally, the potential obstacles and prospects involved in the translational application of these hollow Mn-based nanotheranostics are discussed.

### Single-magnetic-core Mn-based hollow nanoplatforms for MR imaging-guided cancer therapies

#### Hollow Mn_x_O_y_ alone

There are several categories of hollow Mn_x_O_y_ nanomaterials, including HMnO_2_ [93], HMnO [94], HMn_3_O_4_ [95], and HMn_2_O_3_ [96]. HMnO_2_ is the most popular for cancer theranostics. The synthesis of HMnO_2_ often involves a sacrificial template-based method, which uses solid silica nanoparticles and polymer nanoparticles as substrates. After dissolution of the inner cores, the resultant cavity can be used to carry a variety of small molecule drugs, such as chemotherapeutic agents, photosensitizers, and photothermal agents. For chemotherapy, PDT and PTT, respectively.

**Fig. 1:**
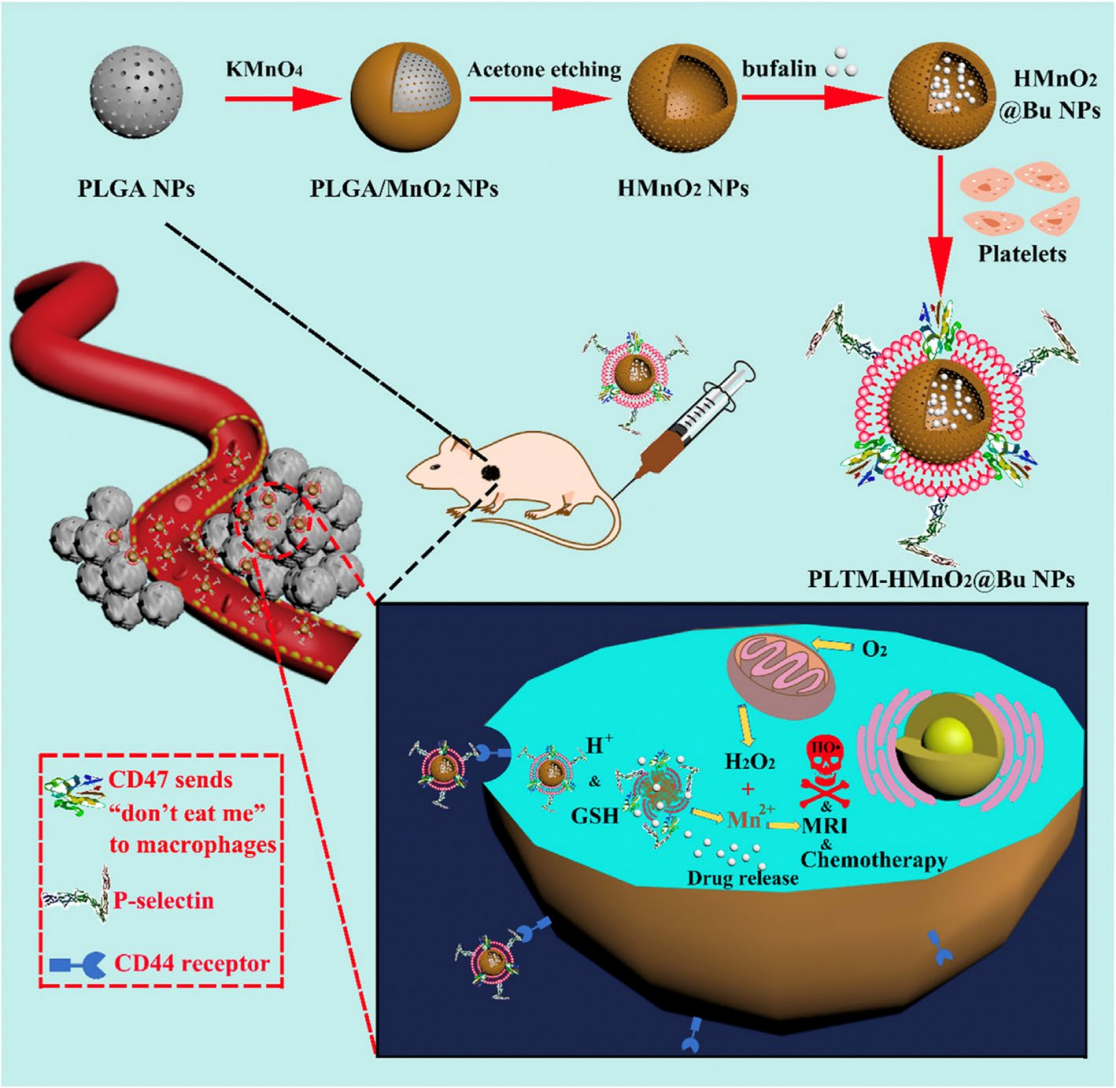
Illustration of the preparation of PLTM-HMnO_2_@Bu NPs and in vivo MR iamging-monitored targeted chemo-chemodynamic combined therapy. Reproduced with permission from Ref. [81]. Copyright 2020, Elsevier Inc.

For example, Wang et al. [81] utilized poly(lactic-co-glycolic acid) (PLGA) nanoparticles as a template to prepare HMnO_2_, which further served as a nanocarrier to deliver bufalin to the tumor site with the help of a platelet membrane (PLTM) (Fig. 1). Platelet modification was able to prevent the phagocytic uptake of the as-prepared PLTM-HMnO_2_@Bu nanoparticles by macrophages due to the self-recognition signals sent by the CD47 membrane protein. In addition, the upregulation of P-selectin on the PLTM facilitated the attachment of platelets to tumor cells through specific binding to the overexpressed CD44 receptors. The HMnO_2_ nanoparticles were rapidly degraded at acidic pH and at high levels of GSH, promoting bufalin (Bu) release and the simultaneous formation of Mn^2+^. The resultant Mn^2+^ further catalyzed the conversion of endogenous H_2_O_2_ to hydroxyl radicals (•OH) for CDT. Tumor growth was effectively inhibited by PLTM-HMnO_2_@Bu nanoparticles due to the combination of CDT and chemotherapy. Additionally, the off-to-on TME-responsive MR imaging performance was examined, and the signal intensities of the tumor site displayed a gradual increase with time. These results revealed that PLTM-HMnO_2_@Bu was very promising for targeted MR imaging guidance and enhanced cancer treatment.

**Fig. 2:**
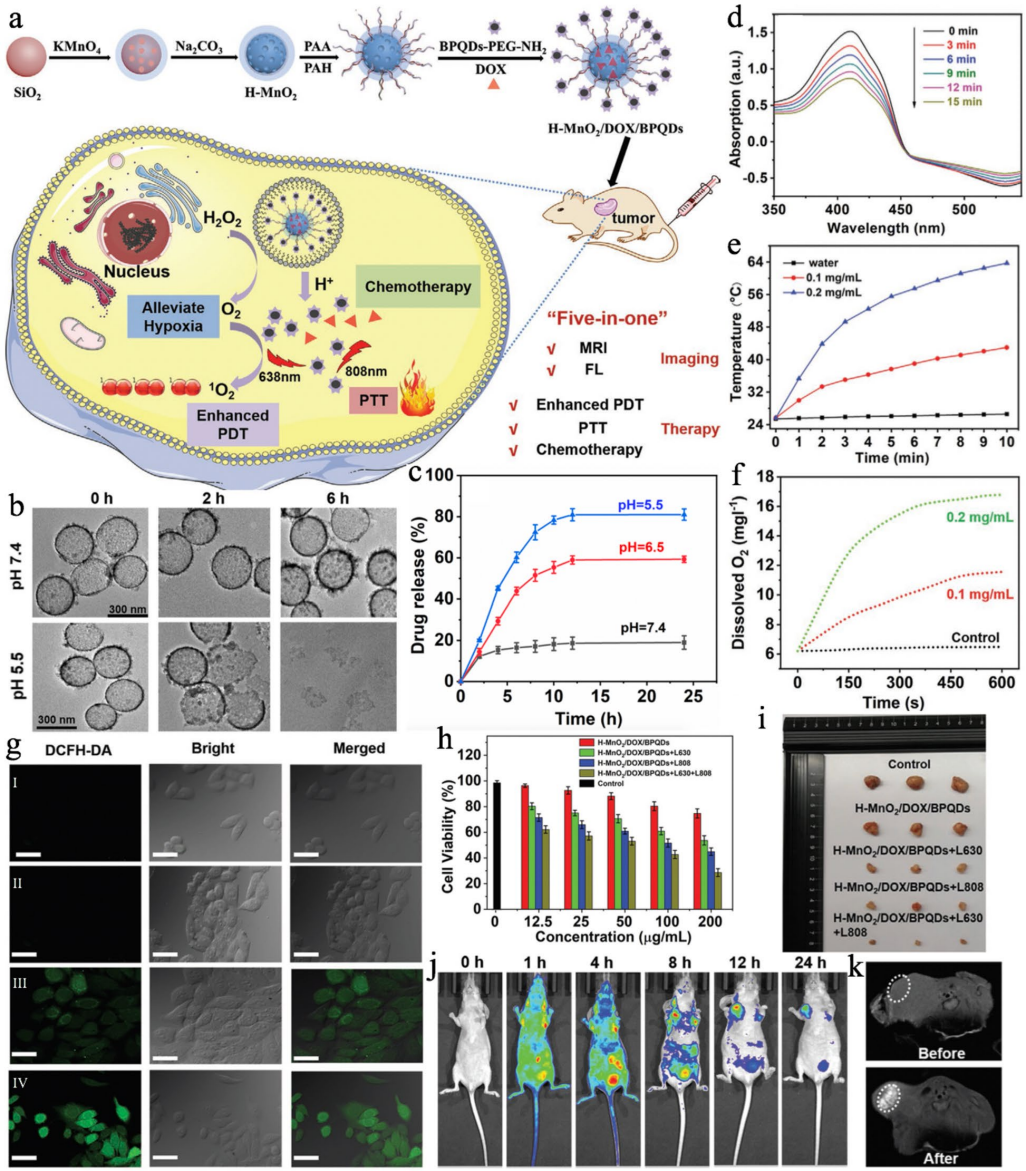
**a** Schematic illustration of nanoprobe H-MnO_2_/DOX/BPQDs synthesis route and its applications for hypoxic cancer multimodal imaging and synergistic therapy, involving MR/FL imaging and enhanced PDT/PTT/chemotherapy. **b** TEM photos of H-MnO_2_/DOX/BPQDs incubated with pH 7.4 (physiological condition) and pH 5.5 buffer (tumor environment) for different times. **c** Percentages of released DOX from H-MnO_2_/DOX/BPQDs over time in the PBS at different pH values (7.4, 6.5, and 5.5). Data were presented as means ± standard deviation (s.d.) (*n* = 3). **d** The ^1^O_2_ generation ability of H-MnO_2_/DOX/BPQDs in air with 1,3-diphenylisobenzofuran (DPBF). **e** Photothermal heating curves of the H-MnO_2_/DOX/BPQDs aqueous dispersions with different concentrations (0, 0.1, and 0.2 mg mL^− 1^) and H-MnO_2_/DOX upon 808 nm laser irradiation (1.0 W cm^− 2^). **f** The dissolved O_2_ concentration in 100 μm H_2_O_2_ solutions after treated with different concentrations of H-MnO_2_/DOX/BPQDs (0, 0.1, and 0.2 mg mL^− 1^). **g** Confocal images of intracellular ROS generation in HepG2 cells treated with Control + L630 (I), H-MnO_2_/DOX + L630 (II), BPQDs-PEG-NH_2_ + L630 (III), and H-MnO_2_/DOX/BPQDs + L630 (IV), as detected with 2′,7′-dichlorodihydrofluorescein diacetate (DCFH-DA). **h** Cell viability of HepG2 cells after treatment with various concentrations of H-MnO_2_/DOX/BPQDs under 630 and/or 808 nm laser irradiation. **i** The photographs of tumor dissection of different treatment groups obtained at 15 days. **j** In vivo fluorescence images of HepG2 tumor-bearing mice at different time points after systemic administration of nanoprobe H-MnO_2_/DOX/BPQDs (MnO_2_: 10 mg kg^− 1^; DOX: 4.5 mg kg^− 1^; BPQDs: 10 mg kg^− 1^) via tail vein injection. **k** In vivo T_1_-MR images of a mouse taken before and after systemic administration of H-MnO_2_/DOX/BPQDs at 24 h. Reproduced with permission from Ref. [93]. Copyright 2021, Wiley-VCH Verlag GmbH & Co. KGaA, Weinheim

To realize more accurate cancer imaging and efficient therapy, Wu et al. [93] reported a multifunctional nanotheranostic (H-MnO_2_/DOX/BPQDs), in which black phosphorus quantum dots (BPQDs) served as both the photosensitizer and photothermal agent. Briefly, monodispersed SiO_2_ nanoparticles, the hard template, were dissolved after in situ decoration of the mesoporous MnO_2_ layer on their surface. Then, the obtained H-MnO_2_ was sequentially modified with poly(allylamine hydrochloride) (PAH) and poly(acrylic acid) (PAA). Subsequently, BPQDs-PEG-NH_2_ (PEG for polyethylene glycol) was covalently grafted onto H-MnO_2_-PAH-PAA by a carbodiimide cross-linking reaction, followed by loading with doxorubicin (DOX) (Fig. 2a). As shown in Fig. 2b, the as-prepared monodispersed H-MnO_2_/DOX/BPQDs were hollow and spherical in shape with an average particle size of ~ 300 nm. The decomposition behaviour was then investigated by incubating H-MnO_2_/DOX/BPQDs in PBS solution at pH 7.4 and pH 5.0. The morphology in the pH 7.4 groups showed no significant changes, while obvious collapses were found in the pH 5.0 groups, indicating the great potential of H-MnO_2_ as a pH-sensitive nanocarrier (Fig. 2c). Upon 630 nm laser irradiation, the absorbance of the H-MnO_2_/DOX/BPQDs and 1,3-diphenylisobenzofuran (DPBF) mixture showed a decrease over time, which indicated singlet oxygen (^1^O_2_) generation due to the presence of BPQDs (Fig. 2d). Moreover, the photothermal performance of the H-MnO_2_/DOX/BPQDs was also demonstrated, as revealed by the temperature elevation when exposed to an 808 nm laser (Fig. 2e). Furthermore, the H-MnO_2_/DOX/BPQDs displayed typical catalase (CAT)-like property, providing sufficient O_2_ for enhanced PDT in the H_2_O_2_ solution (Fig. 2f). Consequently, the H-MnO_2_/DOX/BPQDs + L630 group showed the strongest cellular green fluorescence of ROS. Taking all these features together, the MnO_2_/DOX/BPQDs was expected to be an ideal therapeutic agent. As shown in Fig. 2h, only 28.63% of the HepG2 cells survived after treatment with H-MnO_2_/DOX/BPQDs + L630 + L808 when the concentration was 200 μg mL^− 1^, revealing that the combined chemophototherapy was much more effective than PDT, PTT or chemotherapy alone. Consistently, a significantly higher tumor growth inhibition rate was obtained in the H-MnO_2_/DOX/BPQDs + L630 + L808 group (Fig. 2i). In addition, the strongest fluorescence intensity of DOX in the tumor was observed at 12 h post-injection due to the EPR effect, and the T_1_-MR signals displayed a 4-fold increase after injection for 24 h (Fig. 2j, k). The H-MnO_2_/DOX/BPQDs + L630 + L808 with dual-modal MR/FL imaging and synergistic chemotherapy/PDT/PTT capabilities showed great promise for improving diagnostic accuracy and therapeutic outcomes.

**Fig. 3:**
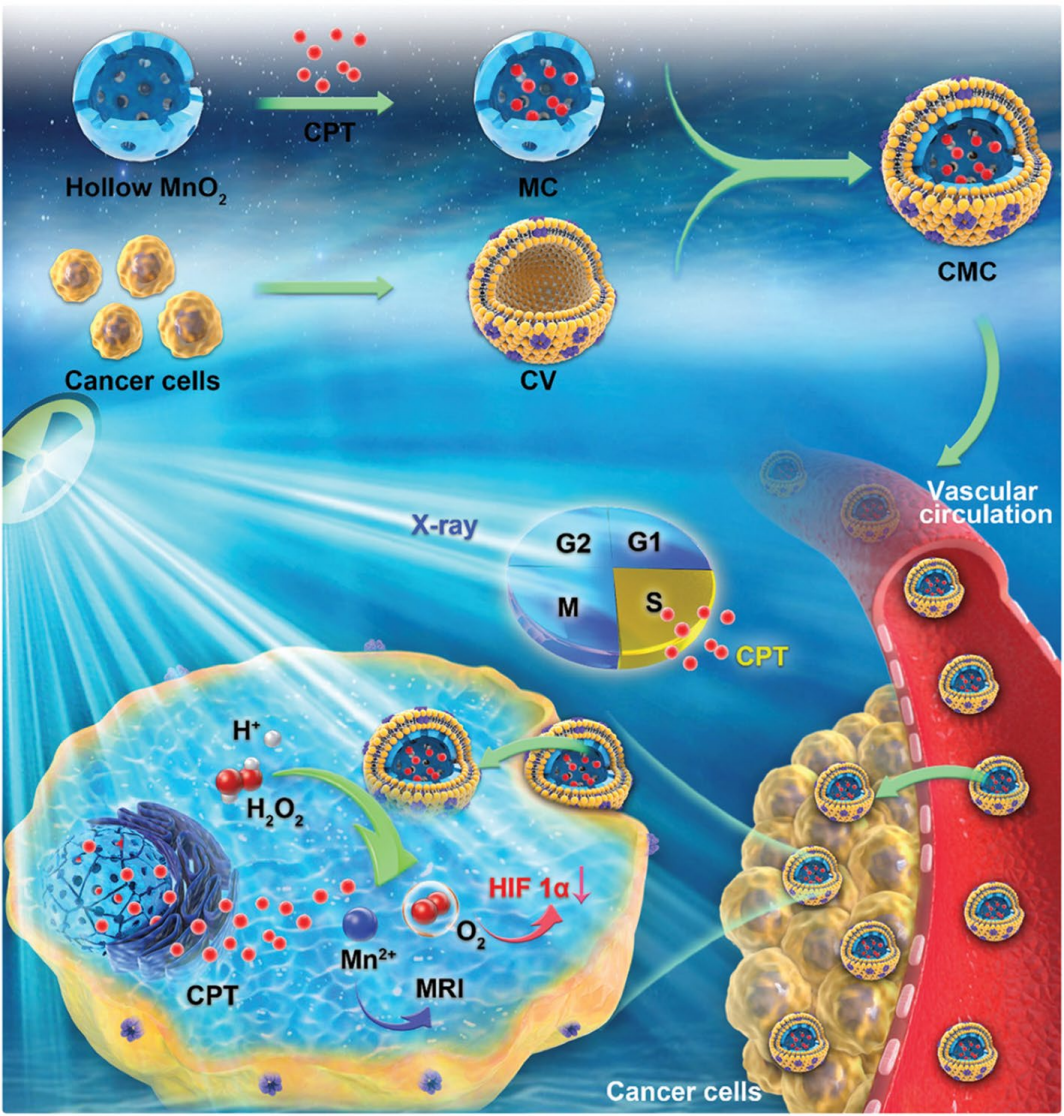
Schematic illustration of the biomimetic nanozyme/CPT hybrid system for synergistically enhanced RT. Reproduced with permission from Ref. [97]. Copyright 2020, The Royal Society of Chemistry

Zhu et al. [97] constructed cancer cell vesicle (CV)-coated HMnO_2_ nanoparticles with camptothecin (CPT) encapsulated (denoted CMC) to boost RT, which was the first example of utilizing HMnO_2_ in RT sensitization (Fig. 3). The cancer cell membrane coating endowed the nanoparticles with prolonged blood circulation and targeting ability. After cellular uptake, HMnO_2_ reacted with the acidic H_2_O_2_ to generate large amounts of O_2_ and release CPT and Mn^2+^ ions. The antitumor mechanism mainly involved the following: (1) O_2_ production was capable of suppressing hypoxia inducible factor-1 (HIF-1) expression, thus improving the RT sensitivity of the cells, and (2) a low dose of CPT blocked the cell cycle in the S-phase (radiosensitive phase), which further promoted radiation-induced damage. Additionally, the released Mn^2+^ acted as a T_1_-weighted MR imaging CA to identify the tumor sites. This work offeres a new idea for designing RT sensitization systems.

As another type of Mn_x_O_y_, MnO is also of widespread interest for cancer imaging and therapy. For example, Wei et al. [98] reported novel octapod-shaped hollow porous manganese(II) oxide (HPMO) nanoparticles with small particle sizes for stimuli-responsive T_1_-weighted MR imaging and targeted cargo delivery. The zwitterionic dopamine sulfonate (ZDS)-modified HPMO nanoparticles were able to be a versatile platform for loading organic dyes or chemotherapeutic drugs. Upon encountering the TME, especially lysosomes, the as-prepared DOX@HPMO could be decomposed into Mn^2+^ ions with cargoes subsequently released. The liberated DOX then recovered its fluorescence that was previously quenched by HPMO, visualizing the release process. Meanwhile, Mn^2+^ could be used for T_1_-weighted MR imaging, which also enabled monitoring the in vivo DOX release in real time. Thanks to the pH-sensitive dual-modal imaging modalities and site-specific drug delivery, this versatile and intelligent nanoplatform was beneficial for accurate cancer diagnosis and effective therapy.

#### Hollow matrix-supported Mn_x_O_y_

MnO_2_ can also be engineered on different hollow matrices, and the intrinsic performances of those matrices together with the features of MnO_2_ are expected to bring about a greater breakthrough in cancer theranostics [99, 100]. As a proof of concept, a hollow matrix possessing photothermal effect or Fenton-like catalytic performance could be used for PTT or CDT, cooperating with the therapeutic features of MnO_2_ to further improve the treatment outcomes [101].

PDA has become a new class of biomaterials for biomedical applications owing to its various biological functions and unique chemical properties [102–104]. In addition to being a good photothermal agent for photoacoustic (PA) imaging/PTT, the plentiful aromatic rings and functional groups of PDA make it possible to load chemical drugs and facilitate surface modification as well as the chelation of diverse metal ions for multi-modal imaging [105–107]. Hollow-structured PDA (HPDA) is regarded as an excellent nanocarrier due to its large cavity and biodegradability in the acidic TME [108, 109]. Wang et al. [71] fabricated a novel cancer-specific and TME-responsive nanoplatform (HPDA@MnO_2_@RGD@Ce6/DOX, denoted as HPMRCD) for FL/MR imaging and combined chemotherapy/PDT. The HPMRCD was enriched in the tumor site as a result of the targeting ability of arginine-glycine-aspartic acid (RGD) and then instantly decomposed in the acidic high-H_2_O_2_ TME. The generated O_2_ could alleviate tumor hypoxia, while the released Mn^2+^ ions led to greatly improved contrast of T_1_-weighted MR imaging. Simultaneously, the degradation of the inner HPDA resulted in the effective release of loaded DOX and Ce6, thus remarkably inhibiting tumor growth by employing dual-modal chemotherapy and PDT. Interestingly, the in vivo fluorescence imaging of Ce6 was also able to locate the tumor site and guide the therapeutic process. This work provides a promising method for chemotherapy/PDT using a self-enhanced theranostic nanoplatform.

The strong surface plasmon resonance (SPR) endows noble metal nanoparticles with prominent optical and photothermal performances, including a high absorption cross-section and superior photothermal conversion efficiency in the NIR biowindow, resulting in extensive application prospects in bioimaging and PTT [110–112]. Based on hollow Au/Ag alloy nanoparticles, Wu et al. [113] fabricated a versatile nanoplatform (Au/Ag-MnO_2_-PEG/Ce6, denoted as AAM-Ce6) with MnO_2_ and PEG functionalized as well as Ce6 loaded. The AAM showed intensive optical absorption in the NIR-II region and possessed remarkable photothermal effects (PCE = 52.5% at 1064 nm) for NIR-II PTT and PA imaging. After AAM-Ce6 reached the tumor site, the outer MnO_2_ nanoparticles quickly responded to the TME, producing a large amount of O_2_ to promote PDT and massive amounts of Mn^2+^ ions to turn on MR imaging. Meanwhile, the released Ce6 also provided decent FL imaging performance and converted O_2_ to ^1^O_2_ for enhanced PDT under a 660 nm laser. When concurrently treated with 1064 nm and 660 nm lasers, AAM-Ce6 exhibited synergistically improved therapeutic efficacy, which was much better than PTT or PDT alone.

**Fig. 4:**
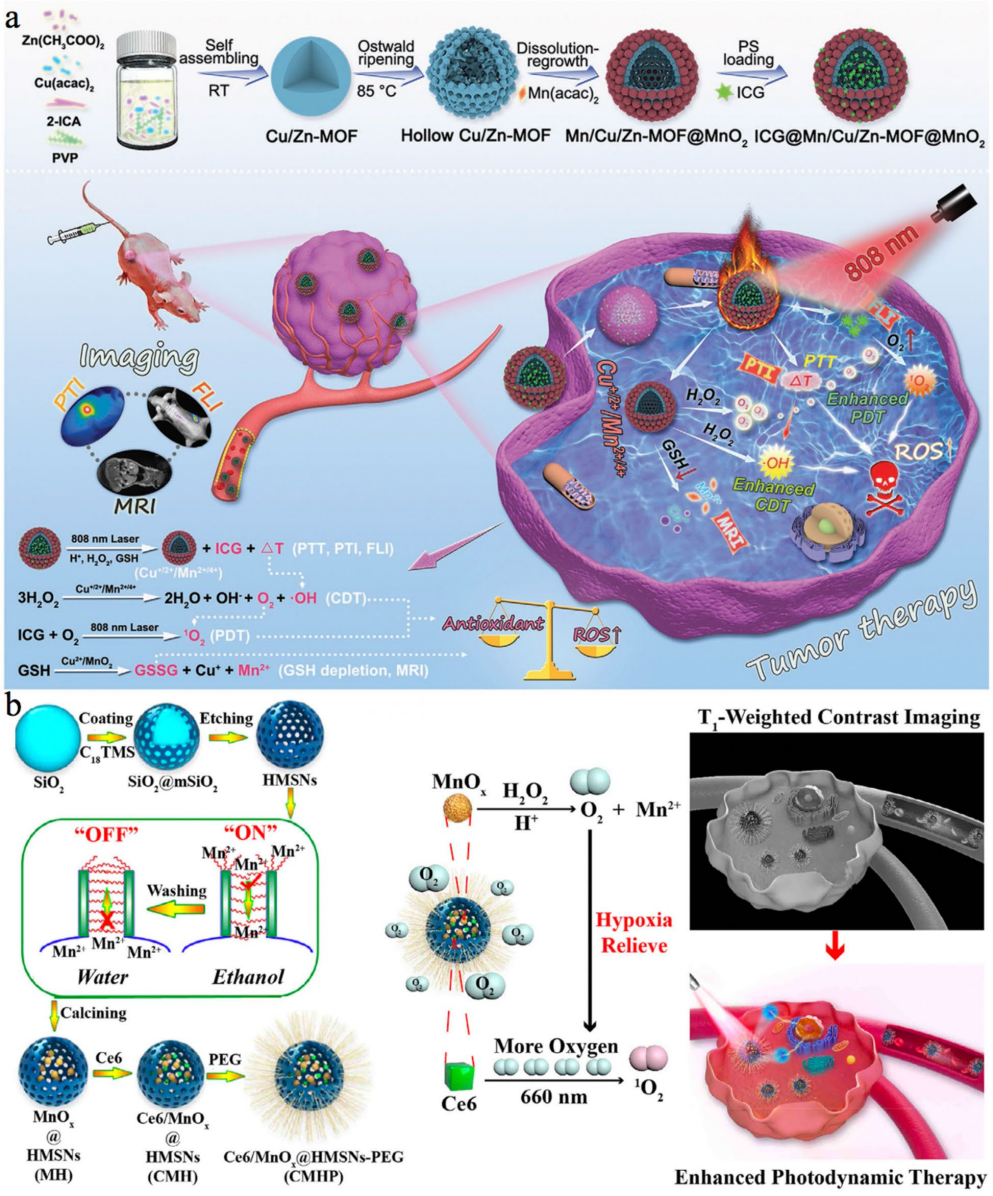
**a** Schematic illustration for the fabrication of ICG@Mn/Cu/Zn-MOF@MnO_2_ and the scheme of therapeutic mechanism for PTI/FL/MR imaging guided ROS-augmented synergistic PTT/PDT/CDT. Reproduced with permission from Ref. [92]. Copyright 2021, Wiley-VCH Verlag GmbH & Co. KGaA, Weinheim. **b** Diagrams of the synthetic protocol and MR imaging-guided PDT of CMHP nanoreactors including detailed synthetic steps of site-specific loading both MnO_x_ and Ce6 into HMSNs based on on/off state of mesoporous channels and schematic diagram of theranostic functions of CMHP nanoreactors, i.e., MR imaging-guided and oxygen generation to improve PDT efficacy. Reproduced with permission from Ref. [117]. Copyright 2020, Elsevier Inc.

MOFs consisting of metal ions and organic ligands hold great potential for theranostic applications due to their channels/pores and active metal ions [114, 115]. In addition, MOFs are also selected as templates or substrates to obtain multifunctionality [87, 116]. Utilizing a mixed-metal Cu/Zn-MOF as the precursor, Cheng et al. [92] proposed a novel hollow nanoplatform (ICG@Mn/Cu/Zn-MOF@MnO_2_) for triple-modal imaging and synergistic PTT/PDT/CDT. The synthetic process of ICG@Mn/Cu/Zn-MOF@MnO_2_ is illustrated in Fig. 4a. Briefly, Cu/Zn-MOF was first prepared and underwent the Ostwald ripening process to obtain a hollow porous structure with coexisting Cu^+^ and Cu^2+^. Subsequently, the manganese(II) acetylacetonate (Mn(aac)_2_) solution was added under heating treatment to introduce Mn^2+^ and MnO_2_, followed by ICG encapsulation. The aggregation of ICG in ICG@Mn/Cu/Zn-MOF@MnO_2_ allowed good photothermal imaging (PTI) and PTT upon exposure to laser irradiation. Along with the degradation of ICG@Mn/Cu/Zn-MOF@MnO_2_, ICG was gradually released, and its FL imaging and PDT capacities were recovered. Moreover, the in situ catalytic decomposition of intratumoral H_2_O_2_ led to massive O_2_ production, enhancing ICG-induced PDT. Furthermore, the Cu^+^ and Mn^2+^ ions were ideal Fenton-like agents that could intensively catalyze H_2_O_2_ to generate highly active and cytotoxic •OH for improved CDT with the assistance of hyperthermia from PTT. Notably, these ROS-mediated therapies were further improved due to the depletion of GSH by Cu^2+^ and MnO_2_. In addition, the TME-activated MR imaging capability was also evaluated for therapeutic guidance. This novel antitumor paradigm achieved highly efficient treatment and caused insignificant damage to normal tissues, realizing the integration of multi-functions onto hollow nanoplatforms for improved diagnosis and therapies via synergistic manners.

Based on the above, most studies have focused on decorating Mn_x_O_y_ on the outside of hollow substrates, but it is uncommon to see Mn_x_O_y_ encapsulated inside the cavity of the matrix. For the first time, Du et al. [117] reported a smart on/off switching method to load both MnO_x_ and Ce6 into hollow mesoporous silica nanoparticle (HMSN) (denoted CMH) (Fig. 4b). The key to this novel system was the utilization of the surfactant C18TMS, a mesostructured-directing agent possessing diverse solubilities in various solvents. When C18TMS-containing HMSNs were dispersed in ethanol, C18TMS was dissolved to open the mesoporous channel (on state); thus, the Mn precursor could easily enter the hollow structures. In contrast, C18TMS showed hydrophobicity in water, which closed the mesoporous channel (off state) and confined the Mn precursor inside. The C18TMS was removed after calcination with MnO_x_ generated, and the channel opened; thus, Ce6 could also be loaded. This new method guaranteed the precise encapsulation of both Mn and Ce6, in which MnO_x_ was capable of decomposing endogenous H_2_O_2_ to produce O_2_ and Mn^2+^ to improve the efficiency of Ce6-induced PDT and T_1_-weighted MR imaging, respectively. This work introduces a novel strategy that enabled confined nanoparticles to grow within hollow structures, paving a new way to design multifunctional nanoplatforms for different biomedical applications.

#### Mn-doped hollow nanoparticles

Metal-doped nanoparticles, another unique type of nanomaterial, are recommended for application in biomedical applications because of their low self-toxicity [118–121]. Doping with Mn enhances the magnetic and catalytic properties of substrates that are in favor of bioimaging and cancer therapy. For example, Mn-doped ZnO_2_ endowed the nanoplatform with activatable MR imaging performance, which could be used to monitor acid-induced ZnO_2_ dissociation and the subsequent treatment process [122]. Moreover, Tian et al. [123] found that Mn incorporation could easily alter the Fe_2_P electron density, significantly promoting Fe catalytic activity with the generation of 4-fold •OH. Furthermore, Fu et al. [124] reported Mn-doped ZrMOF nanocubes displaying highly effective combined microwave dynamic and thermal cancer therapies.

Regarding Mn-doped hollow nanoparticles, Zou et al. [125] developed a pH/GSH dual-responsive theranostic nanoplatform (DOX-Mn-ZGOCS-PEG) based on Mn-doped hollow silica with ultrasmall persistent phosphor (ZGOCS)/DOX co-loaded and PEG modified. In this design, the −Mn−O− bonds were intensively dissociated under acidic and reducing TME, leading to the gradual biodegradation of DOX-Mn-ZGOCS-PEG and massive DOX release. Meanwhile, the resultant Mn^2+^ was able to greatly heighten the contrast of T_1_-weighted MR imaging. More importantly, the previously quenched persistent luminescence (PL) of ZGOCS was recovered for autofluorescence-free diagnosis as DOX-Mn-ZGOCS-PEG disintegrated. In vivo experiments showed that DOX-Mn-ZGOCS-PEG displayed an impressive tumor inhibition rate with negligible side effects and good biodegradability. Together, DOX-Mn-ZGOCS-PEG could realize tumor-targeted boosted chemotherapy under the guidance of TME-activated MR/NIR-PL dual-modal imaging, holding enormous translational potential in accurate cancer diagnosis and therapy.

**Fig. 5:**
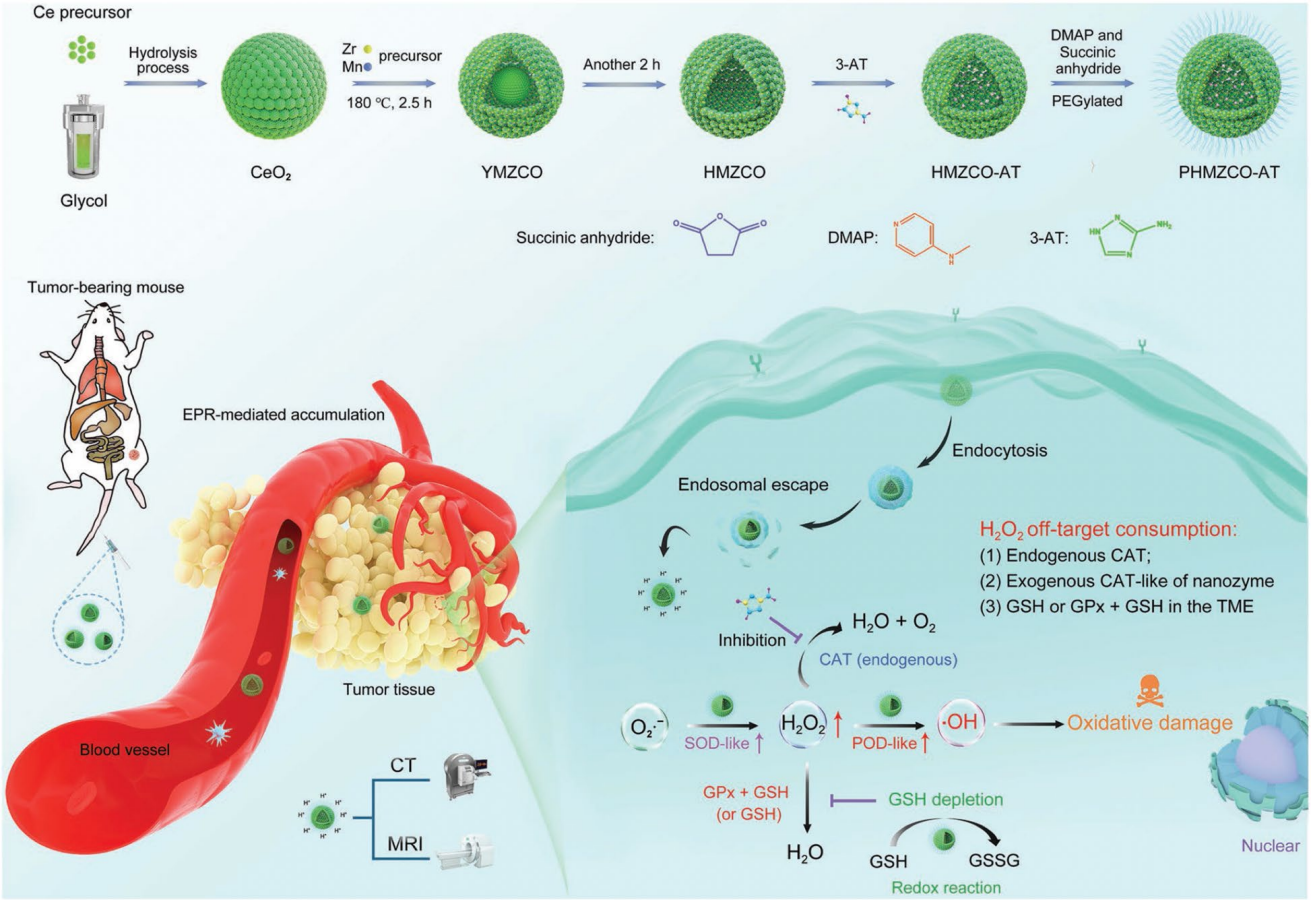
Design, fabrication, and catalysis-based therapeutic schemes of PHMZCO-AT tandem nanozyme including synthetic procedure of PHMZCO-AT nanozymes with hollow or yolk-shell structure and scheme of catalytic H_2_O_2_ generation, inhibition of the off-target H_2_O_2_ consumption, and continuous •OH production for intensive NCDT by PHMZCO-AT nanozymes. Reproduced with permission from Ref. [126]. Copyright 2022, Wiley-VCH Verlag GmbH & Co. KGaA, Weinheim

Dong et al. [126] reported a hollow mesoporous tandem nanozyme (denoted PHMZCO-AT) for T_1_-weighted MR/CT imaging-guided highly efficient catalytic treatment (Fig. 5). By co-doping Mn^4+^/Zr^2+^ into CeO_2_ nanoparticles, a hollow-structured HMZCO was formed based on the Kirkendall effect. Subsequently, 3-amino-1,2,4-triazole (3-AT) was encapsulated into the interior cavity, and PEG was functionalized on the surface to obtain PHMZCO-AT. Compared to the pure CeO_2_ nanoparticles, the as-prepared PHMZCO-AT nanozyme possessed enhanced superoxide dismutase (SOD)- and peroxidase (POD)-like activities under mildly acidic condition because the variable-valence Mn ions doping triggered intermetallic charge transfer and thus accelerated the Ce^4+^/Ce^3+^ redox cycles. In addition, the loaded AT in PHMZCO-AT acted as an endogenous CAT inhibitor and weakened the catalytic decomposition of H_2_O_2_, as evidenced by the elevated Michaelis–Menten constants (*K*_m_ = 180.67 mM) and reduced maximum reaction rate (*V*_max_ = 0.12 mg L^− 1^ min^− 1^) at pH 5.5, while the *K*_m_ and *V*_max_ of pure CeO_2_ at pH 7.4 were 77.64 mM and 0.34 mg L^− 1^ min^− 1^, respectively. When PHMZCO-AT reached the TME, the endogenous superoxide anion (O_2_•^−^) was first converted to H_2_O_2_ due to the SOD-like activity. Then, the POD-like activity of PHMZCO-AT allowed the generation of massive amounts of highly toxic •OH from the elevated H_2_O_2_. Interestingly, the H_2_O_2_ level was further enhanced as a result of the CAT-suppressive feature of the 3-AT molecule and GSH-depleting behavior of PHMZCO-AT, boosting the production of •OH for effective oxidative damage, and a superior tumor inhibition rate (81.9%) was achieved on 4 T1 tumor xenografts. In addition, the paramagnetic property of Mn^2+^ and Zr with high X-ray damping capacity enabled PHMZCO-AT to serve as both a T_1_-weighted MR imaging and a CT imaging CA. This work provides a general strategy to construct advanced nanozymes with artfully modulated multi-enzymatic performances for highly efficient catalytic therapy upon the guidance of multi-modal imaging.

#### Mn complex-based hollow nanoparticles

Loading versatile Mn complexes into hollow structures has also become a research topic of high interest. For example, Yan et al. [127] fabricated an all-in-one nanoplatform (denoted as aHNF) with DOX, Ce6 and Mn^2+^ ions co-encapsulated inside the cavity of hollow silica nanoparticles under mild, eco-friendly and convenient reaction conditions, in which partial Mn^2+^ ions were captured by the drug molecules. Finally, the outermost surface was modified with PEG to endow the nanoformulations with good biocompatibility and prolonged blood circulation. In this way, the multiple treatment units could be transported to the tumor tissues and then synergistically functioned to relieve the therapeutic resistance with enhanced antitumor efficacy. Briefly, the nanosystem improved the bioavailability of Ce6 as well as its release behavior, markedly promoting the PDT outcomes. Furthermore, Mn^2+^ not only was able to serve as a good MR imaging CA for diagnosis but also activated a Fenton-like reaction to generate plenty of •OH as well as amplify the DOX- and Ce6-induced cytotoxicity, such as intracellular oxidative stress elevation and oxidation defense disruption. In vivo experiments indicated the unsatisfactory treatment effects of chemotherapy even with the involvement of Mn^2+^, but the combined group displayed significant tumor inhibition after PDT inclusion as revealed by the remarkably decreased tumor volumes. Additionally, the nanoformulations could be completely degraded in the physiological environment and caused no significant side effects in vitro and in vivo.

**Fig. 6:**
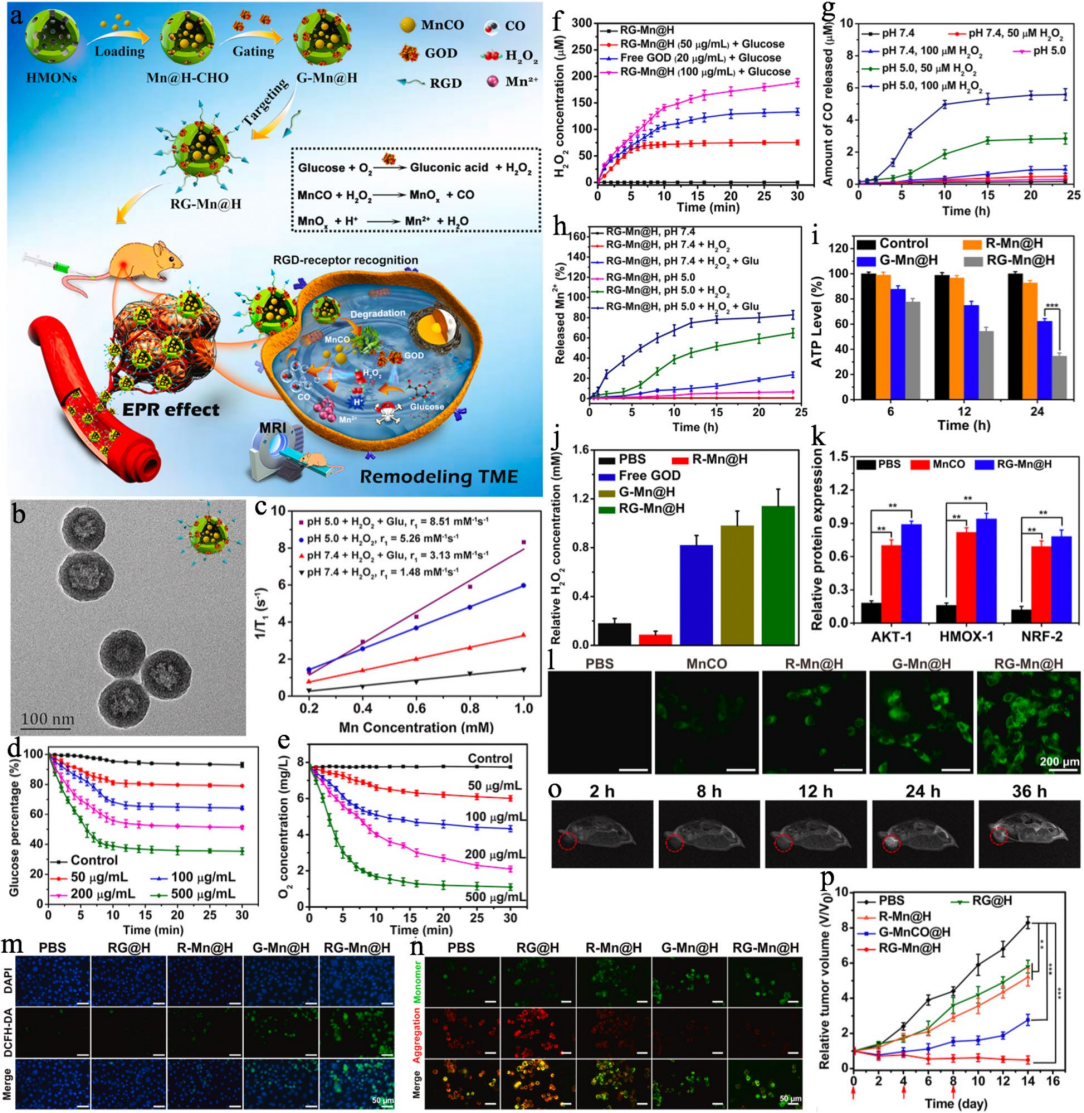
**a** Schematic illustrations of the construction of targeted cascade nanocatalyst (RG-Mn@H) for remodeling TME, the sequential cascade reactions mechanism of RG-Mn@H on the generation of H_2_O_2_ for controllable CO release, and MR imaging-monitored combinatorial therapy of breast cancer. **b** TEM image of RG-Mn@H. **c** Relaxivity fits of RG-Mn@H in PBS solutions (50 μM of H_2_O_2_, pH 5.0 or 7.4) with or without addition of glucose. **d** Glucose concentration and (**e**) Oxygen concentration plotted against time in different concentrations of RG-Mn@H solution with the addition of glucose (10 mM) and H_2_O_2_ (50 μM). **f** The change of H_2_O_2_ concentrations in the glucose solution (10 mM) with the addition of RG-Mn@H (50 or 100 μg/mL) and free GOD (20 μg/mL). **g** In vitro cumulative release of CO from RG-Mn@H at different pH values with different amounts of H_2_O_2_. **h** The release profile of Mn^2+^ from RG-Mn@H (3 mg/mL) in PBS solutions (pH 5.0 or 7.4) with or without addition of glucose (Glu) and H_2_O_2_. **i** ATP level in MDA-MB 231 cells after different treatments as indicated. ***P < 0.001. Error bars indicate standard deviation (*n* = 5). **j** The H_2_O_2_ level in MDA-MB-231 cells after different treatments ([Mn]: 10 μg/mL). **k** Quantitative results of the Akt-1, Nrf-2, and HMOX-1 expression after different treatments. **P < 0.01. Error bars indicate standard deviation (*n* = 3). **l** Intracellular CO level detected by using COP-1 CO fluorescence probe ([Mn]: 10 μg/mL). Fluorescence images of MDA-MB-231 cells after different treatments and stained with (**m)** intracellular DCFH-DA probe and (**n**) JC-1 dye (red, aggregates; green, monomers). **o** In vivo T_1_-weighted MR images of the tumor bearing mice at different time points after i.v. injection of RG-Mn@H (Mn: 5 mg/kg, tumors are indicated by the red rings). **p** The tumor growth curves during different treatments (*n* = 5). Reproduced with permission from Ref. [129]. Copyright 2021, Elsevier Inc.

Manganese carbonyl (MnCO), as a prodrug of CO gas and Mn^2+^possesses bright prospects in cancer theranostics due to the following merits: (1) Gas therapy does not cause drug resistance but is able to sensitize drug-resistant cells to chemotherapeutic drugs. (2) Gas therapy is regarded as a green therapeutic method that induces no significant systemic toxicity or side effects. (3) The resultant Mn^2+^ can act as both a good MR imaging CA and Fenton-like agent [128]. For example, Wu et al. [129] proposed a “TME remodelling” nanoplatform (RGD-GOD-MnCO@HMONs, denoted RG-Mn@H) for in situ continued CO release and cancer-specific MR imaging-guided synergistic gas/starvation therapy (Fig. 6a). The biodegradable HMON cores were the key to this design, which not only encapsulated MnCO but also enabled glucose oxidase (GOD) to grow on the surface. The pH-sensitive GOD acted as a gatekeeper to avoid the premature leakage of MnCO and facilitated further modification with the tumor-targeting unit RGD. The monodispersed RG-Mn@H displayed a hollow spherical morphology with an average particle size of approximately 100.8 nm, and the catalytic behavior of GOD was evidenced by the decrease of glucose/O_2_ level and increase of H_2_O_2_ concentration (Fig. 6b and d-f). The elevated acidity resulted in the dissociation of GOD and subsequently caused the release of the loaded MnCO, which was further decomposed to CO and Mn^2+^ ions by the generated H_2_O_2_ (Fig. 6g, h). These features were systematically investigated at the cellular level, and the ATP level was dramatically decreased in the RG-Mn@H group; however, the corresponding H_2_O_2_ concentration was significantly enhanced that could promote the production of CO (Fig. 6i, j and l). Interestingly, the generated CO was able in turn to boost ROS formation, resulting in the intensive green fluorescence of DCF in the RG-Mn@H group (Fig. 6m). Moreover, the R-Mn@H-, G-Mn@H- and RG-Mn@H-treated cells displayed stronger green fluorescence, indicating the efficient induction of mitochondrial dysfunction by CO (Fig. 6n). Furthermore, western blotting analysis showed the significant up-regulation of Akt-1, Nrf-2, and HMOX-1, which demonstrated that the release of CO activated the Akt signalling pathway to amplify the treatment outcome (Fig. 6k). Notably, tumor growth was markedly and synergistically suppressed after RG-Mn@H treatment, resulting in a much higher inhibition rate (94.3%) than R–Mn@H (inhibition rate = 35.5%) or G-Mn@H (inhibition rate = 66.7%) (Fig. 6p). Additionally, the released paramagnetic Mn^2+^ ions could be used for T_1_-weighted MR imaging (r_1_ = 8.51 mM^− 1^ s^− 1^ in acidic H_2_O_2_/glucose solution) that monitored the treatment process (Fig. 6c and o). This intelligent nanoreactor holds unique potential for cancer-targeted imaging and augmenting gas-induced therapy.

Analogously, Zheng et al. [130] loaded MnCO into the cavity of hollow mesoporous CuS nanoparticles for MR imaging-guided combined PTT/gas therapy. The multifunctional MnCO@CuS was proven to possess good biocompatibility and low toxicity. Once accumulated in tumor tissues, the overproduced H_2_O_2_ could trigger the release of CO from MnCO, which was further accelerated upon NIR irradiation. Meanwhile, the intermediate MnO_x_ was decomposed to Mn^2+^ to allow T_1_-weighted MR imaging (r_1_ = 8.64 mM^− 1^ s^− 1^ when treated with pH 5.5 + 100 μM H_2_O_2_ + NIR) in the presence of acidic TME. In vitro and in vivo experiments demonstrated that MnCO@CuS + Laser exhibited the strongest anticancer effects; only 3.56% of the cells survived, and tumors in this group were almost completely eradicated at 14 days post-injection.

### Dual-magnetic-cores Mn-based hollow nanoplatforms for MR imaging-guided cancer therapies

In addition to Mn, Co- and Fe-based nanomaterials have attracted tremendous attention in the biomedical field [131–135]. Generally, nanomaterials containing Co and Fe are good T_2_-weighted MR imaging CAs and Fenton−/Fenton-like agents due to their intrinsic magnetic and catalytic properties, respectively. In addition, they display great potential in magnetic hyperthermia and PTT, which can synergize with other diagnosis and treatment functions [136–139]. In this section, we will present dual magnetic cores, including Mn-Co-based and Mn-Fe-based nanoplatforms, for cancer theranostics.

**Fig. 7:**
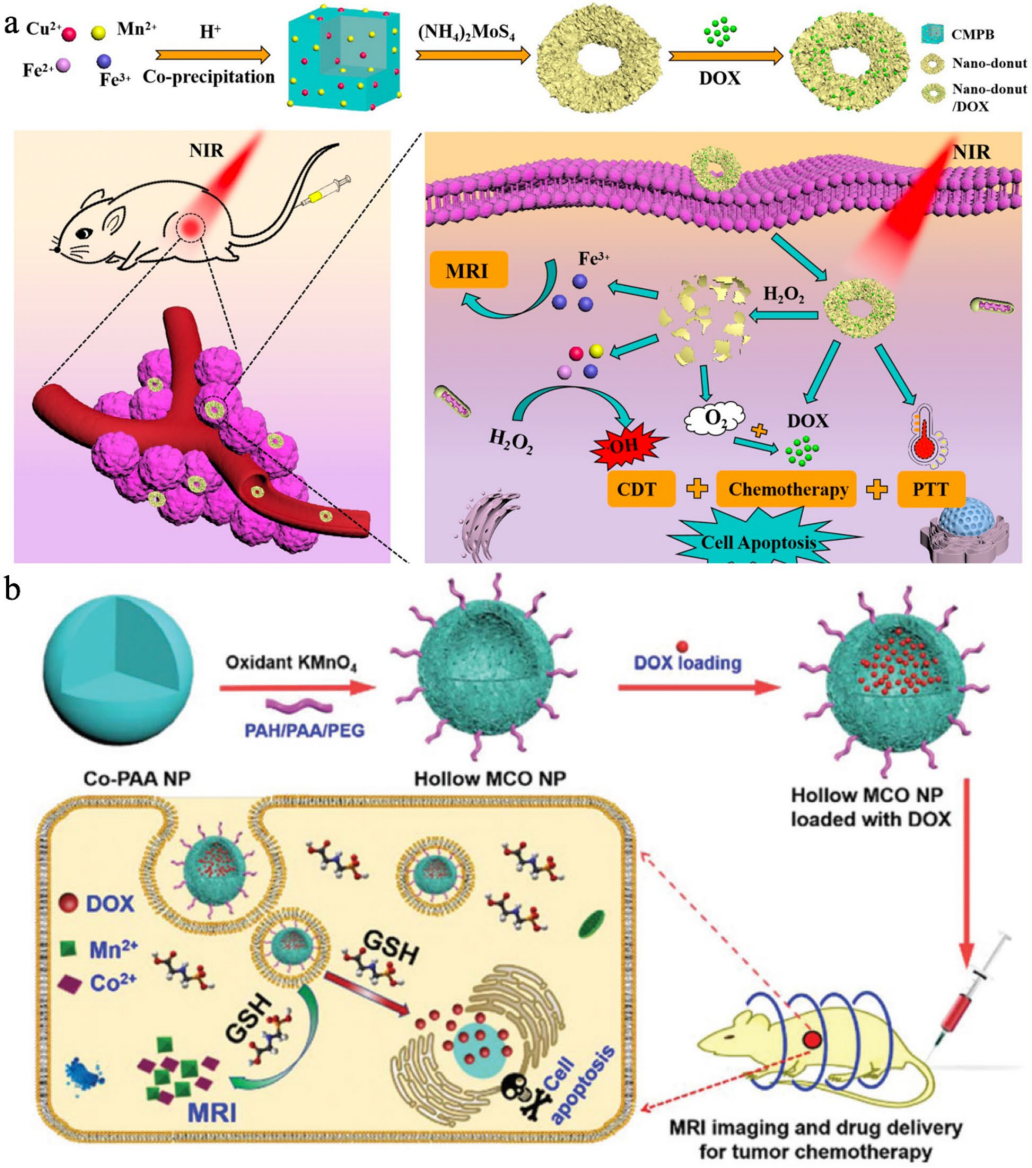
**a** Scheme of the synthesis process and therapeutic mechanism of Nano-donut/DOX nanoplatforms. Reproduced with permission from Ref. [141]. Copyright 2022, Elsevier Inc. **b** The schematic illustration of MCO NP synthesis and their application as GSH-responsive nanoscale DDSs. Reproduced with permission from Ref. [140]. Copyright 2019, The Royal Society of Chemistry

A size-tunable hollow nanoplatform (manganese/cobalt oxide, denoted as MCO NP) was reported by Ren et al. [140] for T_1_-T_2_ dual-modal MR imaging and drug delivery (Fig. 7a). Hollow MCO was synthesized by a one-step redox reaction of PAA-stabilized Co nanoparticles and KMnO_4_. The hollow cavities were formed due to the Kirkendall effect, i.e., the different diffusion rates of MnO_4_^−^ and Co atoms. By varying the PAA amount, the MCO NP could be synthesized with controlled diameters ranging from 50 to 300 nm. Taking the 70 nm-MCO NP with a cavity size of 30 nm as an example, it proved to be a desirable nanocarrier for hydrophilic DOX loading by diffusion into the cavity and electrostatic interactions with PAA. Subsequent experiments demonstrated that the DOX-encapsulated MCO NP acted as both GSH-triggered CAs and DDSs for tumor diagnosis and chemotherapy. In contrast to the intact MCO, the T_1−_ and T_2_-weighted MR imaging signals were obviously enhanced after degradation by GSH, reaching a 2.24- and 3.43-fold increment, respectively. At the same time, effective killing effects towards cancer cells and significant tumor suppression were noticed as a result of the released DOX.

Ferric hexacyanoferrate, also known as Prussian blue (PB), is a biocompatible photothermal agent that has been extensively investigated for cancer therapy, but its single therapeutic function with insufficient photothermal effect still hampers further applications in the clinic [142]. Based on Cu^2+^/Mn^2+^ co-doped PB (CMPB) nanoparticles, Guan et al. [141] fabricated a biodegradable nanoplatform with TME-responsive catalysis for MR imaging and enhanced PTT/CDT/chemotherapy (Fig. 7b). Interestingly, the (NH_4_)_2_MoS_4_ treatment enabled CMPB to form a hollow structure, and the final Nano-donut (CMPB-MoS_2_-PEG) was obtained after PEG modification. Subsequently, DOX was encapsulated into the cavity of the Nano-donut and could be delivered to the tumor site to amplify the therapeutic effects. The Nano-donut showed rapid responsiveness to endogenous H_2_O_2_, leading to decomposition of the framework. The released multivalent elements (Cu/Fe/Mn ions) were able to decrease the bandgap and thus synergistically promote Fenton/Fenton-like reactions for enhanced CDT. Moreover, the presence of Mn^4+^ could also facilitate O_2_ generation by reacting with H_2_O_2_ to alleviate the hypoxic TME, improving the chemotherapeutic efficacy of DOX. Furthermore, the mingling of MoS_2_ and PB significantly enhanced the photothermal conversion efficiency, ranging from 16.02% (PB only) to 38.0%. Additionally, Fe^3+^ was demonstrated to be a decent T_2_-weighted MR imaging CA to guide the treatment process. In vitro and in vivo experiments clearly revealed the remarkable suppressive effects of Nano-donut/DOX on cancer cells and tumors as well as the excellent biological safety.

**Fig. 8:**
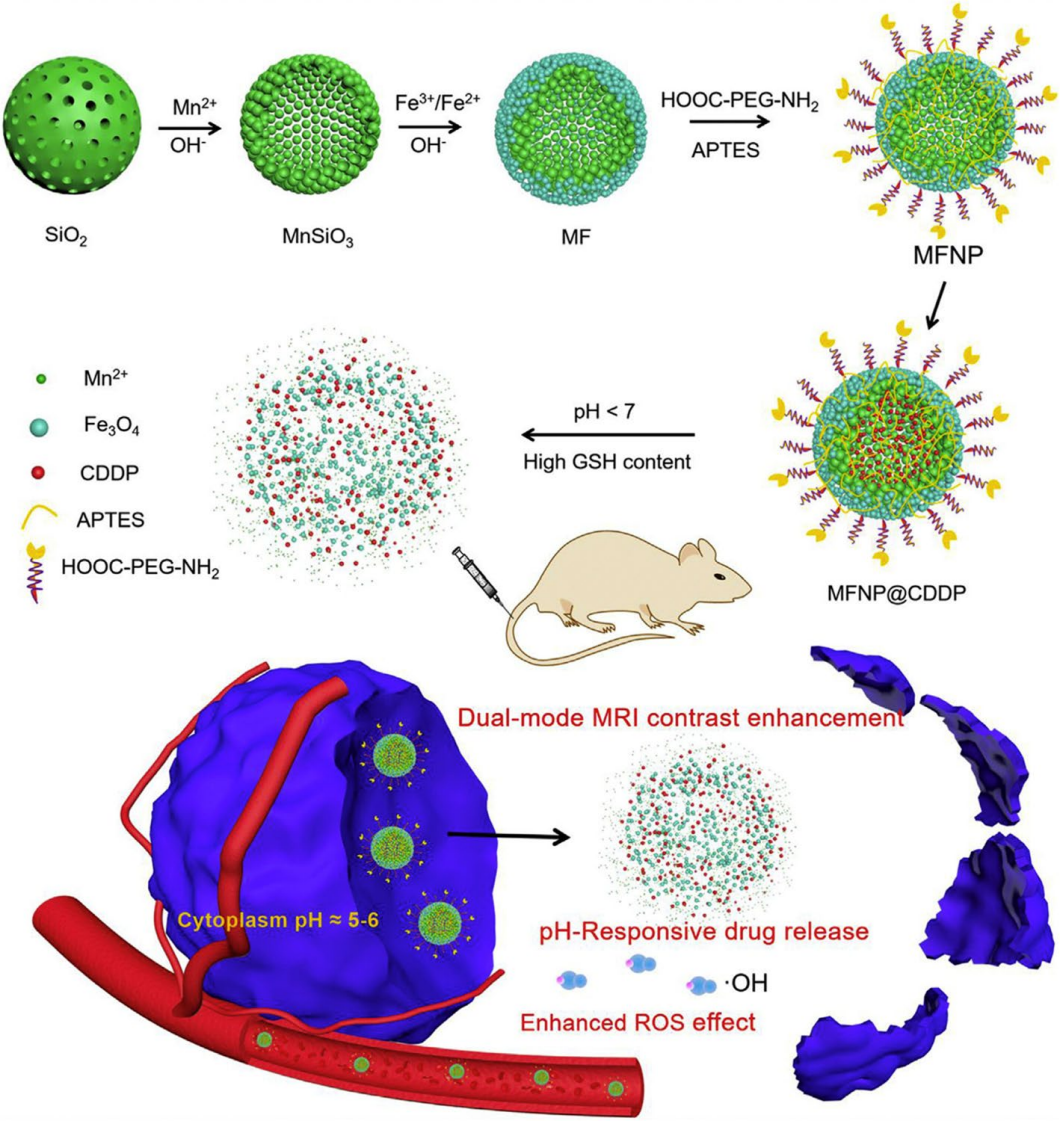
Schematic illustration of the synthetic procedure for MFNP and the nanoplatform integrating dual-mode MR imaging, drug delivery, and ROS generation abilities for cancer diagnosis and therapy. Reproduced with permission from Ref. [145]. Copyright 2022, Elsevier Inc.

Due to the high specific surface area, manganese silicate (MnSiO_3_) can rapidly respond to the weakly acidic and GSH-overproduced TME, acting as a potent T_1_-weighted MR imaging CA and benefiting for drug delivery [143, 144]. In order to improve the diagnostic and therapeutic effects, Sun et al. [145] constructed biodegradable MnSiO_3_@Fe_3_O_4_ (MF) functionalized with PEG (MFNP) and subsequently encapsulated cisplatin (CDDP) to obtain MFNP@CDDP for T_1_-T_2_ dual-modal MR imaging and cooperative cancer treatment (Fig. 8). The decoration of Fe_3_O_4_ nanoparticles on the MnSiO_3_ surface was capable of effectively obstructing the pores of MnSiO_3_ and reducing the premature leakage of loaded CDDP. When the TME was reached, the inner MnSiO_3_ quickly reacted with the weak acid and overproduced GSH, resulting in the collapse of MFNP@CDDP, i.e., the Fe_3_O_4_ nanoparticles separated and CDDP/Mn^2+^ were rapidly released. The resultant Fe_3_O_4_ and Mn^2+^ helped decrease the interference between their T_1_ and T_2_ contrast capabilities, enhancing the dual-modal MR imaging performance (r_1_ = 12.24 mM^− 1^ s^− 1^ and r_2_ = 66.62 mM^− 1^ s^− 1^ at pH 5.5, GSH 10 mM). Additionally, the Fenton-like reaction of Fe_3_O_4_ was boosted during the exfoliation process owing to the increased specific surface area; thus, more highly toxic •OH was generated to induce HeLa cell apoptosis. The therapeutic effects followed the order MFNP@CDDP > CDDP > MFNP, demonstrating the excellent antitumor efficacy of MFNP@CDDP, which was superior to that of CDT or chemotherapy alone.

## Conclusion and perspectives

Over the past decade, the utilization of Mn-based nanoplatforms, especially Mn-based hollow nanoplatforms, has shown promising prospects in cancer theranostics. In this work, we systematically describe the recent advances in Mn-based hollow nanoplatforms, including Mn_x_O_y_, hollow matrix-supported Mn_x_O_y_, Mn-doped hollow nanoparticles, Mn complex-based hollow nanoparticles, hollow Mn-Co nanoparticles and hollow Mn-Fe nanoparticles, for MR imaging-guided therapies. In addition to intrinsic MR imaging and CDT, such hollow nanosystems are also expected to realize synergistic diagnostic and therapeutic effects by rational design and optimization, in which FL imaging, PA imaging, CT imaging, chemotherapy, RT, PTT, PDT, ST and gas therapy can be integrated to compensate for the inadequacies of single-modal diagnosis and treatment. For better comparison, we have summarized the above-mentioned hollow nanoplatforms in terms of materials, templates and mechanisms as well as biomedical applications (Table 1). In short, the encouraging progress in biomedicine presented here brings us much closer to an exciting new paradigm for cancer theranostics. In consideration of the current hurdles and challenges in exploring Mn-based hollow nanoplatforms for clinical applications, our perspectives are as follows:

**Table 1:** Summary of various hollow nanoplatforms for cancer theranostics

Materials	Templates and mechanisms	Biomedical applications	References
PLTM-HMnO_2_@Bu	PLGA	T_1_-weighted MR imaging and CDT/chemotherapy	[81]
H-MnO_2_/DOX/BPQDs	SiO_2_	T_1_-weighted MR/FL imaging and chemotherapy/PDT/PTT	[93]
CMC	SiO_2_	T_1_-weighted MR imaging and chemotherapy/RT	[97]
DOX@HPMO	nanoscale Kirkendall effect	T_1_-weighted MR imaging and chemotherapy	[98]
HPMRCD	SiO_2_	T_1_-weighted MR/FL imaging and chemotherapy/PDT	[71]
AAM-Ce6	galvanic replacement reaction	T_1_-weighted MR/PA//FL imaging and PTT/PDT	[113]
ICG@Mn/Cu/Zn-MOF@MnO_2_	Ostwald ripening process	T_1_-weighted MR/FL/PT imaging and PTT/PDT/CDT	[92]
CMH	SiO_2_	T_1_-weighted MR imaging and PDT	[117]
DOX-Mn-ZGOCS-PEG	SiO_2_	T_1_-weighted MR/NIR-PL and chemotherapy	[125]
PHMZCO-AT	nanoscale Kirkendall effect	T_1_-weighted MR/CT imaging and CDT	[126]
aHNF	calcium carbonate	T_1_-weighted MR imaging and chemotherapy/PDT	[127]
RG-Mn@H	SiO_2_	T_1_-weighted MR imaging and ST/gas therapy	[129]
MnCO@CuS	Ostwald ripening process	T_1_-weighted MR imaging and PTT/gas therapy	[130]
CMPB-MoS_2_-PEG	Ostwald ripening process	T_2_-weighted MR imaging and PTT/CDT/chemotherapy	[141]
MFNP@CDDP	SiO_2_	T_1_−/T_2_-weighted MR imaging and CDT/chemotherapy	[145]
DOX-encapsulated MCO	nanoscale Kirkendall effect	T_1_−/T_2_-weighted MR imaging and chemotherapy	[140]

(1) Safety is one of the most important concerns for clinically translational nanomedicine. Desirable nanotheranostics should exhibit non-toxicity at normal physiological environments but recover their imaging features and generate a large number of therapeutic species for various treatments. Although MnO_2_ has been proven to possess low toxicity and good biocompatibility biodegradability, the introduced substances and hollow matrix may raise the risk of toxicity. More detailed biological and biosafety assessments of these Mn-based hollow nanoplatforms are in urgent need, and their potential risks should be further evaluated, adding chronic toxicity evaluation to the current acute toxicity assessments. In addition, the biodistribution, excretion and potential harm towards specific organs also need to be explored. At present, most in vivo experiments are carried out on mice, and large animal models such primates, should be updated to better investigate the toxicities in the body.

(2) Cancer-specific units, including platelets, cancer cell membranes and RGD have been adopted in these Mn-based hollow nanoplatforms, but the majority of them are decorated with PEG and other polymers, such as polyvinyl pyrrolidone (PVP) or ZDS, to improve the physiological stability, biocompatibility and blood circulation time. Further studies should focus on the development of active targeting nanoplatforms, aiming to facilitate considerable tumor accumulation and promoting diagnostic and therapeutic effects. Notably, the fabrication of organelle-targeted nanoplatforms has been regarded as one of the hottest topics in recent years [146–149]. Triphenyl phosphonium (TPP), a typical mitochondrial targeting molecule, favours ROS/gas-mediated therapies [150–152]. For instance, a multifunctional nanotheranostic based on TPP-modified hollow CuS was reported to integrate hypoxia-activated chemotherapy, PDT and PTT for synergistically treating cancer and maximizing the therapeutic biowindow [153]. Other organelles, such as the nucleus and lysosome, are also good targets for constructing highly effective cancer-specific theranostics [154, 155].

(3) Notably, multi-modal treatment modalities could significantly enhance curative outcomes, and more efforts should be made to explore synergistic manners rather than simply combining them. For instance, PTT has been demonstrated to increase the oxygen flow and the catalytic reaction rates, which is beneficial for PDT, CDT, etc. In light of these, the photothermal agents that are incorporated into the hollow structures should be equipped with desired PCE and photostability. In addition, the laser wavelength used for PTT should be extended to the NIR-II region and even farther away.

(4) The size of the hollow nanoplatforms and the cavity volume should be adjusted to increase tumor accumulation and drug-loading efficiency, respectively. In addition, a facile and mild synthetic strategy for mass production of hollow nanoplatforms is of great significance before clinical applications. Compared to the sacrificial template-based method, the self-templating method seems to be more appealing as a result of the simple preparation process and reduced chemical waste formation.

Although many issues remain unresolved, we believe that Mn-based hollow nanoplatforms will reach their full potential for translation from bench to bedside with future advances in materials science, chemistry, physics, and medicine.

## Data Availability

Data sharing is not applicable to this article as no datasets were generated or analysed during the current study.
